# The relationship between mobile phone addiction, bedtime procrastination, and campus-based physical activity: a longitudinal follow-up study based on a college student population

**DOI:** 10.3389/fpsyg.2025.1690574

**Published:** 2025-12-10

**Authors:** Mingcai Deng, Zhi Li

**Affiliations:** 1 School of Physical Education, Sichuan Technology and Business University, Meishan, Sichuan, China; 2 Sichuan Water Conservancy Vocational and Technical College, Chengdu, China

**Keywords:** adolescents, mobile phone addiction, bedtime procrastination, campus-based physical activity, cross-lagged analysis

## Abstract

**Objective:**

To examine the relationship between mobile phone addiction (MPA), bedtime procrastination (BP), and campus-based physical activity (CBPA) among university students, and to determine whether a “vicious circle” mechanism contributes to health risks, thereby providing a foundation for developing good habits and promoting the overall physical and mental health of university students.

**Design:**

Using the cross-lagged panel model (CLPM) and AMOS 26.0, the analysis was carried out in three steps: “controlling for stability effects - analyzing cross-time predictive paths—testing model fit” to explore the cross-time predictive pathways and mechanisms of action among MPA, BP, and CBPA, and to examine the longitudinal interaction relationships among these variables.

**Methods:**

Two longitudinal follow-up surveys of 376 university students at 3-month intervals using the Mobile Phone Addiction Scale, the Bedtime Procrastination Scale, and the Physical Activity Rating Scale (2025/03/10-2025/06/13).

**Results:**

(1) T1 MPA was significantly and positively correlated with T1 BP (*r* = 0.277, *p* < 0.01) and significantly and negatively correlated with T1 CBPA (*r* = –0.319, *p* < 0.01); T2 MPA was significantly and positively correlated with T2 BP (*r* = 0.433, *p* < 0.01) and significantly and negatively correlated with T2 CBPA (*r* = –0.339, *p* < 0.01). (2) The effect of T1 MPA on both T2 BP (β = 0.27) and T2 CBPA (β = –0.17) was significant (*p* < 0.01). T1 BP on T2 MPA was significant (β = 0.27, *p* < 0.01), and T1 CBPA on T2 BP was significant (β = –0.17, *p* < 0.01).

**Conclusion:**

(1) There is preliminary longitudinal evidence that MPA and BP can mutually provide for each other and form a “vicious cycle” mechanism. (2) There is preliminary longitudinal evidence that MPA provides for CBPA. (3) There is preliminary longitudinal evidence that CBPA provides for BP.

## 1 Introduction

In recent years, factors influencing physical activity among adolescents and issues related to their physical and mental development have garnered attention in many countries, with creating a supportive environment for exercise emerging as a shared consensus (Abera et al., 2021; Lo et al., 2015). However, due to factors such as the widespread adoption of smart devices, shifts in lifestyle habits, and individual cognitive biases, the current state of physical exercise among adolescents in schools remains far from satisfactory. This issue is particularly pronounced among college students (Grasdalsmoen et al., 2020). According to survey data, 48.19% of college students engage in physical activity less than three times per week, and 58.7% of college students exercise for no more than 30 min per session (Daily, 2023a). Chronic physical inactivity not only increases the risk of cardiovascular disease (Dewi et al., 2013), metabolic disorders, and cancer (Chen et al., 2023), but also impairs academic performance and quality of life, posing potential threats to future career development. Therefore, as the primary arena for shaping university students’ personalities and fostering their comprehensive development, higher education institutions must thoroughly investigate the key factors influencing students’ physical activity on campus and the underlying mechanisms at play. This endeavor is not only an urgent necessity for addressing current health challenges but also serves as the theoretical foundation for building a healthy campus ecosystem.

Among the various factors influencing college students’ physical activity, mobile phone addiction has emerged as a key predictive variable. Existing research consistently indicates that mobile phone addiction negatively impacts individuals’ behavioral engagement (Al-Abyadh et al., 2024; Kim et al., 2015). At its core, it is a manifestation of failed self-regulation, referring to a behavioral pattern where excessive mobile phone use disrupts daily life, studies, and interpersonal relationships (Hao et al., 2019). From a behavioral perspective, based on the Self-Regulatory Resource Model, an individual’s self-control capacity is finite. Smartphone addiction continuously depletes cognitive and behavioral resources, leading to diminished regulatory capacity for goal-directed behaviors such as physical activity (Baumeister et al., 2018). From a psychological mechanism perspective, Self-Determination Theory (SDT) further indicates that behavioral implementation is influenced by attitudes, subjective norms, and perceived behavioral control (Ajzen, 2011). Mobile phone addiction not only diminishes individuals’ positive attitudes toward physical activity but also disrupts their time management and execution intentions, thereby reducing their perceived control over exercise participation (Xu et al., 2025). Although studies from perspectives such as Self-Determination Theory (SDT) have suggested that physical activity may alleviate smartphone addiction (Li et al., 2023), the existence of a bidirectional mechanism between smartphone addiction and physical activity requires further validation.

Beyond smartphone addiction, sleep procrastination—another manifestation of failed self-regulation—is also closely linked to physical activity. Procrastination refers to the deliberate delay of task initiation or completion despite anticipating negative consequences (Kadzikowska-Wrzosek, 2020). Surveys indicate that over 90% of college students experience varying degrees of procrastination, with 60.6% exhibiting sleep procrastination. Additionally, 77% of college students struggle to fall asleep on time due to academic pressure and the influence of electronic devices (Daily, 2023b). Regarding the relationship between sleep procrastination and physical activity, the time-bound self-regulation theory emphasizes the influence of time management skills on behavior. Sleep procrastination leads to imbalanced time planning, which crowds out time for physical activity (Dorina et al., 2024; Barz et al., 2014). The Theory of Planned Behavior (TPB) suggests that regular physical activity can alleviate sleep procrastination through three mechanisms: attitude (improving sleep quality), subjective norm (strengthening identification with health-conscious groups), and perceived behavioral control (enhancing self-regulation efficacy) (Fitzgerald et al., 2022; Peluso and Guerra de Andrade, 2005; Schöndube et al., 2017). Regarding the link between sleep procrastination and smartphone addiction, the self-regulation failure theory posits that excessive smartphone use before bed exacerbates sleep procrastination (Magalhaes et al., 2021). Habit theory proposes the opposite possibility: once sleep procrastination becomes ingrained, the resulting time window makes phones—due to their accessibility and entertainment value—the preferred way to fill that void, thereby increasing the risk of addiction (Choi, 2019). Although existing research has preliminarily revealed an association between mobile phone use and sleep problems (Ma et al., 2024), some scholars have also employed longitudinal tracking to uncover the complex interplay among mobile phone use, self-regulation, and health behaviors (Xiao et al., 2025). However, the underlying mechanisms linking mobile phone addiction, sleep procrastination, and physical activity remain unclear, particularly lacking empirical investigations that integrate perspectives on self-regulation and planned behavior.

In summary, mobile phone addiction, sleep procrastination, and physical activity at school appear to form a complex network rather than simple pairwise relationships. While existing research has explored some associations, core questions remain unresolved. First, the direction of prediction—whether bidirectional cross-temporal prediction exists among these factorsmporal prediction exists among these within the school setting. Second, the cyclical mechanism—whether mobile phone addiction and sleep procrastination form a mutually reinforcing vicious cycle. Third, the integrated pathway—how these three factors interact to create a composite health risk pattern.

**FIGURE 1:**
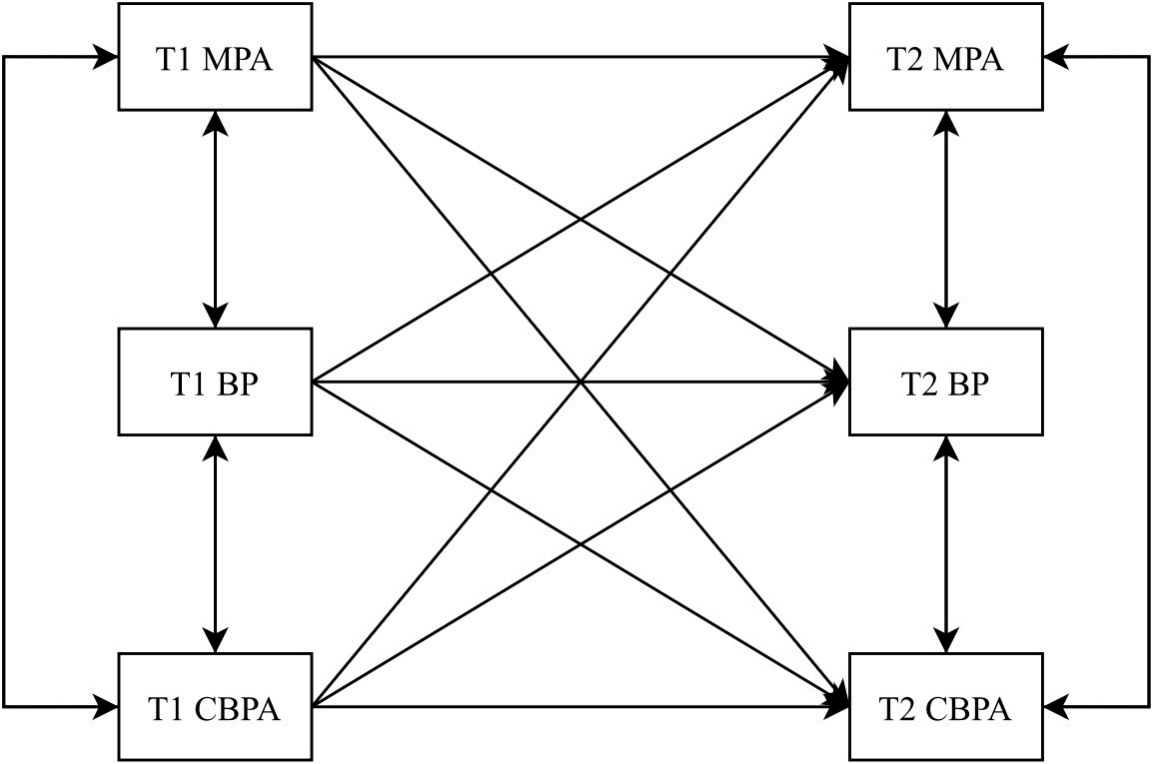
Cross-lagged structural model diagram.

Based on this, the present study employs a longitudinal cross-lagged design targeting university students, focusing on campus settings. It constructs a structural equation model (SEM) of mobile phone addiction, sleep procrastination, and physical activity on campus (Figure 1), hypothesizing a cross-temporal predictive relationship among these three factors. The aim is to provide longitudinal evidence for clarifying the existence of a vicious cycle and revealing the formation mechanism of a composite health risk pattern.

## 2 Materials and methods

### 2.1 Participants

Since most Chinese youth groups in junior and senior high schools mainly board at school and can only use mobile phones on weekends due to the study environment, this study selected a group of college students who can freely use mobile phones. It was conducted with the help of counselors from each class. Following the principle of stratified sampling, three regions of Province A—namely, eastern, central, and western—were chosen based on geographical differences. One comprehensive college was randomly selected in each region to conduct a 3-month longitudinal follow-up survey. Counselors were trained online on the survey process and key points before the questionnaires were distributed. The procedures for both administrations remained the same, with online questionnaires used each time. Before each survey, counselors explained the study’s purpose and use, stressed that participation was voluntary, and allowed participants to withdraw at any point. They also informed participants about how the survey data would be stored and assured confidentiality.

This study employed G*Power 3.1 software to estimate the sample size for the present investigation (Faul et al., 2007). Results indicated that with α set at 0.05, β set at 0.80, and the effect size set at 0.15, the recommended sample size was 98. The first data were collected in March 2025 (T1), and 398 responses were received; the second data collection occurred in June 2025 (T2), with 387 responses. Exclusion criteria such as “regularity of filling in the questionnaire” and “wrong/missing the last six digits of the school number” were applied, resulting in a final valid sample of 376 subjects—those who completed the questionnaire at two times. Of these, 180 (47.9%) were male students and 196 (52.1%) were female students, with an average age of 20.09 ± 1.18 years.

### 2.2 Methods

#### 2.2.1 Mobile phone addiction scale for college students

Adoption of the Smartphone Addiction Scale for College Students developed by Shuang et al. (2014) and Wu et al. (2025a). The scale is based on a 5-point Likert scale, with each entry having a 5-point rating (1 = strongly non-compliant; 2 = non-compliant; 3 = unsure; 4 = compliant; 5 = strongly compliant), for a total of 22 entries. The higher the score, the more serious the mobile phone addiction, and the validation factor analysis showed that the 6-factor conceptual structure was well fitted, which proved that the scale had good structural validity. In this study, the Cronbach’s alpha coefficients for the two tested time points were 0.964 and 0.950, while the results of the validated factor analyses for the two time points showed that: χ^2^/*df* = 1.103, GFI = 0.947, CFI = 0.996, IFI = 0.996, TLI = 0.996, RMSEA = 0.017 (Test1); χ^2^/*df* = 1.490, GFI = 0.931, CFI = 0.975, IFI = 0.975, TLI = 0.973, RMSEA = 0.036 (Test2); this indicates that the scale has good reliability in this study.

#### 2.2.2 Bedtime procrastination scale

The scale adopted is the Chinese version of the Bedtime Procrastination Scale developed by Kroese and revised by Zhang et al. (2021). Primarily designed for college students, it consists of 9 items scored on a 5-point Likert scale (1 = Never; 2 = Rarely; 3 = Sometimes; 4 = Often; 5 = Always). Four items are reverse-scored. Higher scores indicate more severe sleep procrastination tendencies. In the original study, the Cronbach’s alpha coefficient was 0.835, with a test-retest reliability of 0.72, demonstrating high criterion-related validity. In this study, the Cronbach’s alpha coefficients for T1 and T2 were 0.918 and 0.883, respectively. The results of confirmatory factor analysis at the two time points were as follows: χ^2^/*df* = 1.184, GFI = 0.982, CFI = 0.997, IFI = 0.997, TLI = 0.996, RMSEA = 0.022 (Test1); χ^2^/*df* = 1.134, GFI = 0.983, CFI = 0.997, IFI = 0.997, TLI = 0.996, RMSEA = 0.019 (Test2), therefore, the scale had good reliability in this study.

#### 2.2.3 Physical activity rating scale

The Physical Activity Level Scale developed by Liang Deqing (1994) was adopted. This scale is used by domestic scholars to measure college students’ campus-based physical activity (CBPA) (Liang Deqing, 1994; Wu et al., 2025b). It is divided into three dimensions: intensity of physical exercise, duration, and frequency of exercise. The scale uses the Likert 5-point scoring method, and the product of the scores of the three dimensions is used as the total score of physical activity. The activity levels are divided into small-volume exercise (0–19 points), medium-volume exercise (20–42 points), and large-volume exercise (≥ 43 points). In this study, the Cronbach’s α coefficients at time points T1, T2 were 0.828, 0.688, respectively.

#### 2.2.4 Data analysis

In this study, SPSS 27.0 and AMOS 26.0 were used to process and analyze the collected data. Common method bias test, descriptive statistics, correlation analysis, independent samples *t*-test, analysis of variance, and confirmatory factor analysis were carried out through SPSS. AMOS was used to construct the model, examine the relationships between variables, and test the autoregressive coefficients and cross-lag path coefficients (β) of the model.

### 2.3 Common method bias test

Common method bias test based on Harman’s one-factor test criteria (Hao, 2004). The analysis results showed that three factors with an eigenvalue > 1 were extracted at both the first time point (T1) and the second time point (T2). The first factor at each time point explained 39.96 and 36.23% of the total variance, respectively, which are less than the critical threshold of 40%, indicating no serious common methodological bias in this study.

## 3 Results

### 3.1 Descriptive statistics of MPA, BP, and CBPA

Among the measurement tools, based on the formulae of the Mobile Phone Addiction Scale and the Bedtime Procrastination Scale, the theoretical mean values were 55 and 22.5 points, respectively. The Physical Activity Rating Scale was divided into three levels: small, medium, and large. The results of descriptive statistics showed that the mean scores of mobile phone addiction at T1, T2 were 67.90, 68.21, respectively; the scores of bedtime procrastination at T1, T2 were 27.91, 31.23, respectively; and the scores of campus-based physical activities at T1, T2 were 38.60, 38.63, respectively. Thus, the level of mobile phone addiction and bedtime procrastination of college students is at a middle to high level, and the campus-based physical activity is at a middle to low level.

Additionally, using SPSS 27.0, independent-samples *t*-tests for gender and a one-way ANOVA for educational stage were conducted on the three variables across the two assessments. In the independent-samples *t*-tests for gender, Levene’s test for equality of variances was non-significant (*p* > 0.05), indicating that the data satisfied the assumption of homogeneity of variances. Consequently, results assuming equal variances were adopted (Table 1). The *t*-tests for mean equality revealed no statistically significant gender differences in mobile phone addiction, sleep procrastination, or physical activity at school across the two assessments (all *p* > 0.05). Further analysis of effect sizes (Cohen’s d) revealed that all variables exhibited relatively high effect sizes (ranging from 7.731 to 31.290). Although statistical tests failed to reach significance, these findings suggest potential practical differences between genders. This warrants further investigation in subsequent research, considering factors such as sample size. Univariate analysis of variance conducted across educational stages revealed no significant differences in mobile phone addiction, sleep procrastination, or physical activity at school between the two tests (all *p* > 0.05). The effect sizes (η^2^) for these analyses were all low (ranging from 0.001 to 0.026), classified as small effects according to Cohen’s (1988) criteria. This indicates that the proportion of variance in the variables explained by the educational stage factor is very limited, suggesting a minimal practical influence on mobile phone addiction, sleep delay, and physical activity at school (Table 2).

**TABLE 1:** Independent samples *t*-test for gender in T1, T2, and T3.

			Levine’s test of variance equivalence		Mean equivalence *t*-test		Effect size
Grouping variable			*F*	*P*	*t*	*P*	Cohenble z
Gender	T1 MPA	Assuming equal variance	1.739	0.188	–0.468	0.64	18.762
	T1 BP	Assuming equal variance	0.463	0.497	–0.43	0.667	7.939
	T1 CBPA	Assuming equal variance	0.049	0.825	–0.115	0.909	31.290
	T2 MPA	Assuming equal variance	1.252	0.264	–0.09	0.928	17.622
	T2 BP	Assuming equal variance	1.093	0.297	–0.631	0.529	7.731
	T2 CBPA	Assuming equal variance	0.016	0.901	–0.966	0.335	28.859

**TABLE 2:** ANOVA for grade for T1, T2, and T3.

Grouping variables	Implicit variable	Mean square	*F*	*P*	η^2^
Grade	T1 MPA	0.659	0.906	0.460	0.010
	T1 BP	1.189	1.541	0.190	0.016
	T1 CBPA	1.624	1.344	0.110	0.026
	T2 MPA	0.197	0.306	0.874	0.003
	T2 BP	0.035	0.051	0.995	0.001
	T2 CBPA	0.96	1.092	0.360	0.017

### 3.2 Analysis of the correlation between MPA, BP, and CBPA

In this study, the stability test showed that T1 MPA was positively correlated with T2 MPA (*r* = 0.439, *p* < 0.01); T1 BP was positively correlated with T2 BP (*r* = 0.300, *p* < 0.01); T1 CBPA was positively correlated with T2 CBPA (*r* = 0.270, *p* < 0.01). Simultaneous correlation tests showed that T1 MPA was significantly and positively correlated with T1 BP (*r* = 0.277, *p* < 0.01) and significantly and negatively correlated with T1 CBPA (*r* = –0.319, *p* < 0.01); T2 MPA was significantly and positively correlated with T2 BP (*r* = 0.433, *p* < 0.01) and significantly and negatively correlated with T2 CBPA (*r* = –0.339, *p* < 0.01). It was shown that MPA, BP, and CBPA satisfy stability and synchronous correlation across time over a 12-week period, which meets the preliminary conditions for cross-lagged model construction (Table 3).

**TABLE 3:** Mean, standard deviation, and correlation analysis of mobile phone addiction, bedtime procrastination, and physical activity behavior among university students.

Variables	T1 MPA	T1 BP	T1 CBPA	T2 MPA	T2 BP	T2 CBPA
T1 MPA	1	1	1	1	1	1
T1 BP	0.277**					
T1 CBPA	−0.319**	−0.432**				
T2 MPA	0.439**	0.379**	−0.305**			
T2 BP	0.360**	0.300**	−0.334**	0.433**		
T2 CBPA	−0.231**	−0.210**	0.270**	−0.303**	−0.255**	
M	67.90	27.91	38.60	68.21	31.23	38.64
SD	18.74	7.93	31.25	17.60	7.72	28.84

“*” indicates *p* < 0.05, “**” indicates *p* < 0.01.

### 3.3 Cross-lagged analysis of MPA, BP, and CBPA

Structural equation modeling of MPA, BP, and CBPA among university students through AMOS 26.0. Using the method of maximum likelihood (ML), the fitted indicators are shown: χ^2^/*df* = 4.968 (<5), GFI = 0.967 (>0.900), CFI = 0.937 (>0.900), IFI = 0.939 (>0.900), RMSEA = 0.097 (<0.10), indicating a good fit of the model indicators (Table 4).

**TABLE 4:** Indicators of model fit.

Model	χ^2^/df	GFI	CFI	IFI	RMSEA
M2	4.968	0.967	0.937	0.939	0.097
Standard	<5	>0.900	>0.900	>0.900	<0.10

χ^2^/*df*, Chi-square divided by degrees of freedom; RMSEA, Root Mean Square Error of Approximation; GFI, Goodness-of-Fit Index; IFI, Inflated Fit Index; CFI, Comparative Fit Index.

**FIGURE 2:**
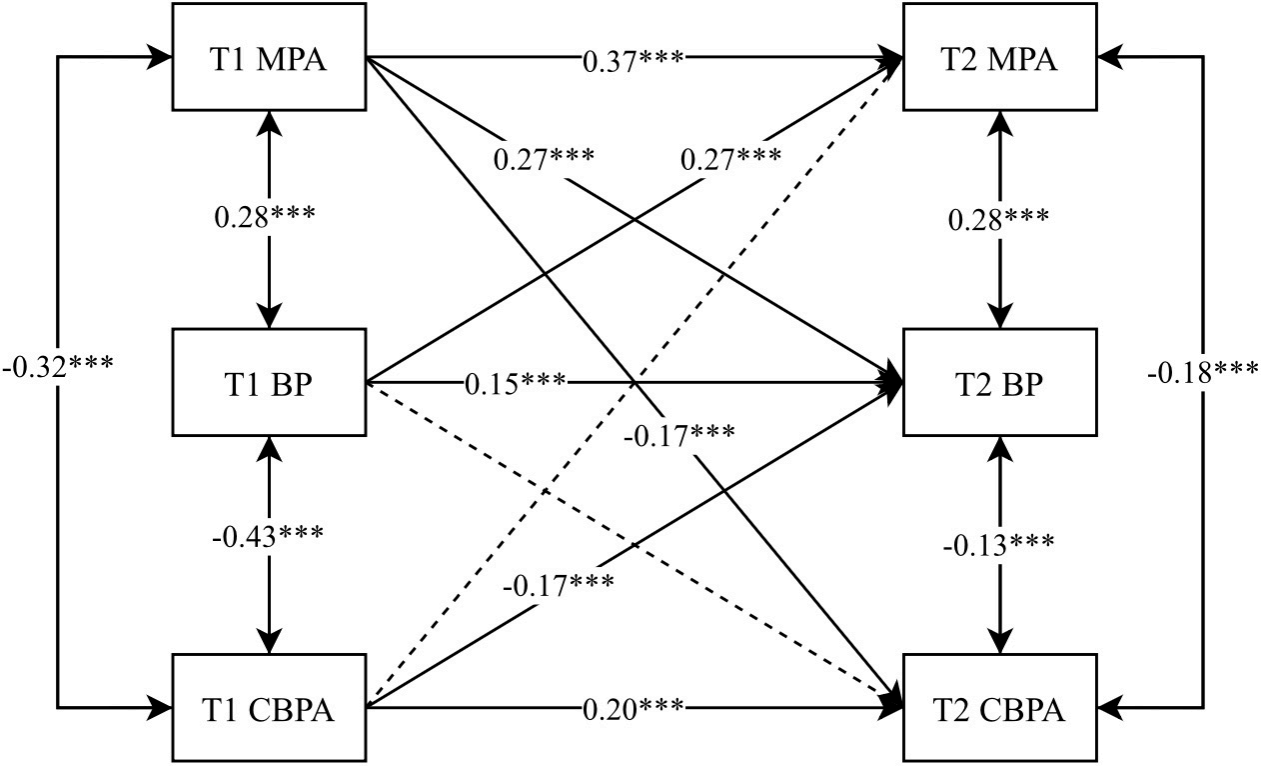
Cross-lagged analysis of MPA, BP, and CBPA. “***” indicates *p* < 0.001 and dashed lines represent non-significant paths.

Combining cross-lagged model path coefficients to examine the asynchronous correlations between MPA, BP, and CBPA. The effect of T1 MPA on both T2 BP (β = 0.27) and T2 CBPA (β = –0.17) was significant (*p* < 0.001); T1 BP on T2 MPA was significant (β = 0.27, *p* < 0.001); and T1 CBPA on T2 BP was significant (β = –0.17, *p* < 0.001) (Figure 2). Combined with cross-lagged path coefficients, this suggests that MPA and BP can predict each other, with MPA negatively predicting CBPA across time, and CBPA negatively predicting BP across time.

## 4 Discussion

### 4.1 General discussion of MPA, BP, and CBPA

This study found that college students exhibit moderately high levels of mobile phone addiction and sleep procrastination, coupled with relatively low levels of physical activity on campus. This pattern reveals a health risk model characterized by “high screen usage—low restorative behaviors—weak exercise participation.” With the widespread adoption of smartphones, they have brought convenience in communication and information access (Reyes, 2016). However, this has also led to increased dependence on mobile phones among college students, particularly manifested in uncontrollable online and short video usage behaviors (Stuckey et al., 2017). From the perspective of enhanced sensitivity theory, intermittent high rewards provided by mobile phone use continuously activate the individual’s behavioral approach system (BAS) while simultaneously inhibiting the behavioral inhibition system (BIS) (Park et al., 2013). In this study, moderate-to-high mobile phone addiction scores indicate BAS hypersensitivity, which continuously reinforces nighttime “phone scrolling” behavior and amplifies delayed discounting effects, thereby leading to sleep procrastination. Concurrently, moderate-intensity physical activity fails to effectively activate BIS regulation of long-term health goals, exacerbating systemic imbalance. Furthermore, the self-control resource model further indicates that daytime academic tasks and multitasking with mobile phones significantly deplete self-control resources; at night, individuals engage in “compensatory sleep deprivation” to restore a sense of control (Baumeister et al., 2019). Low-to-moderate levels of physical activity fail to serve as self-regulatory training, unable to promote resource recovery or growth, thereby making it difficult to break the vicious cycle of “resource depletion—sleep delay” (Luten et al., 2019).

Regarding group differences, whilst this study found no statistically significant differences in mobile phone addiction, sleep procrastination, or physical activity across gender and educational stage, an in-depth analysis of effect sizes provides richer insights into interpreting these findings. Regarding gender differences, the larger Cohen’s d values presented by the independent samples *t*-test (ranging from 7.731 to 31.290) suggest that there may be a trend toward practically significant differences between genders. This finding creates a certain tension with the conjecture that “the widespread advocacy of gender equality in modern society may have diminished the gender-based variability of the study variables.” The substantial effect size indicates that, although not statistically significant in the current sample, the influence of gender may not be entirely negligible. Future research should therefore explore this further using larger samples or more refined behavioral dimensions. By contrast, the effect sizes for stage-of-education factors (η^2^ ranging from 0.001 to 0.026) were all small. This provides strong quantitative support for the interpretation that “undergraduate students exhibit greater homogeneity in educational background and stage of physical and psychological development, hence showing no significant grade-level differences in these health behaviors. “These findings collectively demonstrate that when designing health behavior interventions, it is essential to consider not only statistical significance but also the magnitude of effect size to make a comprehensive assessment. Future interventions may, based on universal strategies, preliminarily consider gender-sensitive designs where appropriate, while the need for grade-specific universal interventions may be relatively less pronounced.

### 4.2 A discussion of the cross-lagged relationship between MPA, BP, and CBPA

This study employed cross-lagged analysis to confirm predictive relationships among mobile phone addiction, sleep procrastination, and on-campus physical activity among college students. Specifically, mobile phone addiction and sleep procrastination mutually predict each other across time, creating a vicious cycle in students’ campus lives. Mobile phone addiction negatively predicts on-campus physical activity across time, while on-campus physical activity predicts sleep procrastination across time.

Previous studies have predominantly treated mobile phone addiction as an independent variable and sleep procrastination as a dependent variable. This study integrates mobile phone addiction, sleep procrastination, and physical activity into a unified longitudinal model, revealing the cross-temporal predictive pathways among these variables. Mobile phone addiction and sleep procrastination can predict each other, supporting an integrated explanation of self-control resource theory and compensatory internet use theory (Kardefelt-Winther, 2014; Vohs et al., 2013). On one hand, smartphone addiction continuously depletes self-control resources, weakening an individual’s ability to inhibit pre-sleep phone use and leading to sleep procrastination (Kroese et al., 2014). On the other hand, daytime fatigue and difficulties in emotional regulation caused by sleep deprivation may prompt individuals to seek emotional compensation through mobile phone use, further exacerbating addictive tendencies (Snodgrass et al., 2018).

Mobile phone addiction exerts a cross-temporal negative predictive effect on physical activity, a finding that can be jointly explained by the time displacement hypothesis and the self-control resource model. First, smartphone use not only crowds out time for physical activity, but the resulting self-depletion also reduces individuals’ willingness and ability to participate in sports activities (Röhlke, 2025). Secondly, when college students develop smartphone addiction, they tend to prioritize phone usage, with screen time occupying their leisure hours and reducing physical activity (Geng et al., 2021). Finally, embodied cognition theory suggests that prolonged static screen-based postures and low levels of proprioceptive input diminish individuals’ implicit associations with the pleasure of movement, thereby weakening their motivation to engage in physical activity (Meier et al., 2012).

Additionally, physical activity at school can predict sleep procrastination negatively across time. According to the Theory of Planned Behavior, an individual’s behavioral intention serves as the primary predictor of actual action, influenced by attitudes, subjective norms, and perceived behavioral control (Ajzen, 2011). Thus, an individual’s positive attitude toward a particular behavior strengthens their intention to perform it. Similarly, the positive subjective norms perceived from society or significant others also reinforce behavioral intentions. When this positive attitude interacts with positive subjective norms, individuals experience heightened perceived behavioral control over performing the behavior. This enhanced perceived behavioral control, in turn, further amplifies their behavioral intentions (Yzer, 2012). It follows that the higher the level of enthusiasm college students have for physical exercise, the more likely they are to avoid sleep procrastination.

### 4.3 Limitations of the study and practical interventions

This study examined the intrinsic predictive relationships among MPA, BP, and CBPA through longitudinal tracking surveys and cross-lagged model design and analysis. However, certain limitations exist, and future research could be improved in the following aspects: (1) The research sample could be expanded further. In subsequent studies, increasing the sample size and surveying college students from different majors could further explore the influence of this demographic variable; (2) Beyond mobile phone addiction and sleep procrastination, attention should be paid to other factors potentially influencing college students’ physical activity on campus. Future research should incorporate additional variables for consideration (Zang et al., 2025); (3) The cross-lagged panel model (CLPM) employed in this study fails to distinguish between within-individual effects and between-individual effects. Future research may adopt more advanced models, such as the random intercept cross-lagged panel model (RI-CLPM), to reveal the dynamic interactions of variables more precisely at the individual level, thereby strengthening causal inference.

At the practical level, universities can develop intervention strategies across the following dimensions to promote the establishment of healthy behavioral patterns among students:

(1) Create a “rhythm-friendly” campus environment to reduce phone dependency and sleep procrastination.

Implement a “Quiet Hours Initiative” in dormitories during designated evening periods, encouraging students to store phones centrally in public study areas or activity rooms to minimize screen exposure before bed. Provide complementary non-screen sleep aids such as relaxation music and mindfulness guidance audio to help students establish a “digital detox” routine 1 h before sleep.

(2) Establish a “low-barrier, high-reinforcement” physical activity mechanism to enhance participation motivation and self-control.

Design a campus fitness points system based on metrics such as steps taken, exercise duration, and participation in programs. Link these points to physical education grades and merit-based awards. Reinforce participation motivation through real-time feedback and periodic rewards (e.g., redeemable for cultural products or sports equipment), thereby restoring immediate positive feedback for physical activity.

(3) Integrate dual-module cognitive-behavioral educational content to enhance students’ self-regulation competencies

Incorporate the self-control resources theory and time substitution mechanisms into mental health and career planning courses. This helps students understand the psychological mechanisms behind smartphone addiction and sleep procrastination while providing behavioral strategies (e.g., task decomposition, goal setting, attention diversion) to strengthen their self-regulation abilities in daytime tasks and nighttime behaviors.

## 5 Conclusion

Longitudinal tracking surveys and cross-lagged model design and analysis explored the mechanisms underlying college students’ mobile phone addiction, sleep procrastination, and physical activity on campus. The findings revealed mutual cross-temporal prediction between mobile phone addiction and sleep procrastination, with mobile phone addiction negatively predicting physical activity on campus across time, and physical activity on campus negatively predicting sleep procrastination. These findings hold practical significance for universities in developing sleep quality improvement programs and addressing mobile phone addiction among college students.

## Data Availability

The original contributions presented in the study are included in the article/supplementary material, further inquiries can be directed to the corresponding author.
